# MicroRNA-873 inhibits colorectal cancer metastasis by targeting ELK1 and STRN4

**DOI:** 10.18632/oncotarget.24115

**Published:** 2018-01-02

**Authors:** Chuannan Fan, Biyu Lin, Zhengjie Huang, Dan Cui, Minyi Zhu, Zhenling Ma, Yunda Zhang, Fan Liu, Yingfu Liu

**Affiliations:** ^1^ State Key Laboratory of Cellular Stress Biology, Innovation Center for Cell Signaling Network, School of Life Sciences, Xiamen University, Fujian Sheng, People’s Republic of China; ^2^ Medical College of Xiamen University, Department of Basic Medical Sciences, Medical College, Xiamen University, Fujian Sheng, People’s Republic of China; ^3^ Department of Surgical Oncology, First Affiliated Hospital of Xiamen University, Fujian Sheng, People’s Republic of China

**Keywords:** colorectal cancer, miR-873, ELK1, STRN4, metastasis

## Abstract

MicroRNAs (miRNAs) are a group of small non-coding RNAs that directly bind to the 3ʹ-untranslated-region (3ʹUTR) of mRNA, thereby blocking gene expression post-transcriptionally. Accumulating evidence prove that microRNA-873 (miR-873) functions as a promoter or suppressor in various cancers, while whether it affects the progression of colorectal cancer (CRC) is yet unknown. Here we found that miR-873 was downregulated in human CRC clinical samples, mouse CRC specimens and cell lines with high metastatic potential. We also demonstrated that low miR-873 expression was closely associated with poor prognosis of CRC. Overexpressing miR-873 suppressed proliferation and metastasis of CRC cells both *in vitro* and *in vivo*, while inhibiting miR-873 expression promoted the proliferation, migration and invasion *in vitro*. Moreover, miR-873 exerted its function by perturbing the ERK-CyclinD1 pathway and the epithelial-mesenchymal transition (EMT) process. Furthermore, we revealed that miR-873 acted as a tumor-suppressive microRNA by directly binding to the 3ʹUTRs of ELK1 and STRN4 and suppressed their expression. Our study uncovered an inhibitory role of miR-873 in CRC progression and might provide a promising marker for CRC diagnosis and prognosis.

## INTRODUCTION

Colorectal cancer (CRC) is one of the most common malignancies. It is the fourth leading cause of cancer mortalities in men and the third one of cancer mortalities in women [1]. The death rate has decreased by approximately 3% every year during the past decades. However, the metastatic lesion formed by primary tumor is still a major cause of CRC death [2]. Although increasing evidence has uncovered diverse regulatory mechanisms involved in CRC progression, pivotal drivers and inhibitors related to this process are largely unknown.

Genomic studies shows that misregulation of TGF-β, WNT and EGFR signaling pathways are general events in CRC progression [3–6]. CRC with chromosomal instability (CIN) and microsatellite instability (MSI) is more inclined to acquire hypermethylation of key genes, which leads to gain higher invasive ability and eventually form macrometastases [7, 8]. MiRNAs are a large class of non-coding RNAs that participate in the regulation of mRNA translation or/and stability by directly binding to target mRNAs. Dysregulation of miRNAs results from genetic or epigenetic modifications is corroborated to be common in the invasion-metastasis cascade of CRC [9, 10].

MiR-873, a miRNA located on the chromosome 9, has been identified as a tumor suppressor in glioblastoma [11], ovarian cancer [12] and breast cancer [13]. However, it has been proved to be an oncogene in lung adenocarcinoma [14]. Of note, whether miR-873 affects CRC progression is yet unclear. Interestingly, a previous study proves that the promoter of miR-873 was widely hypermethylated in most CRC cell lines, which implies that miR-873 may be a potential tumor suppressor in CRC [15]. In this study, we aimed to interrogate the expression characteristics of miR-873 and elucidate the functional importance of it by *in vitro* and *in vivo* methods. In our study, miR-873 was downregulated in CRC patient samples and two mouse CRC models. Furthermore, miR-873 level was inversely correlated to the metastatic potential of clinical specimens and CRC cell lines. Overexpressing miR-873 inhibited CRC cell proliferation, migration and invasion *in vitro*, while inhibition of miR-873 increased CRC cell proliferation, migration and invasion. MiR-873 exerted its tumor-suppressive role by inhibiting the ERK-CyclinD1 axis and the EMT process. We further demonstrated that miR-873 suppressed CRC cell growth and liver metastasis *in vivo*. Very interestingly, we found that ELK1 and STRN4 were direct targets of miR-873. In conclusion, miR-873 functions as a vital molecular participant in CRC growth and metastasis and our data may shed light on further use of miR-873 as a new diagnostic and prognostic biomarker of CRC.

## RESULTS

### MiR-873 may act as a tumor suppressor in CRC

**Figure 1:**
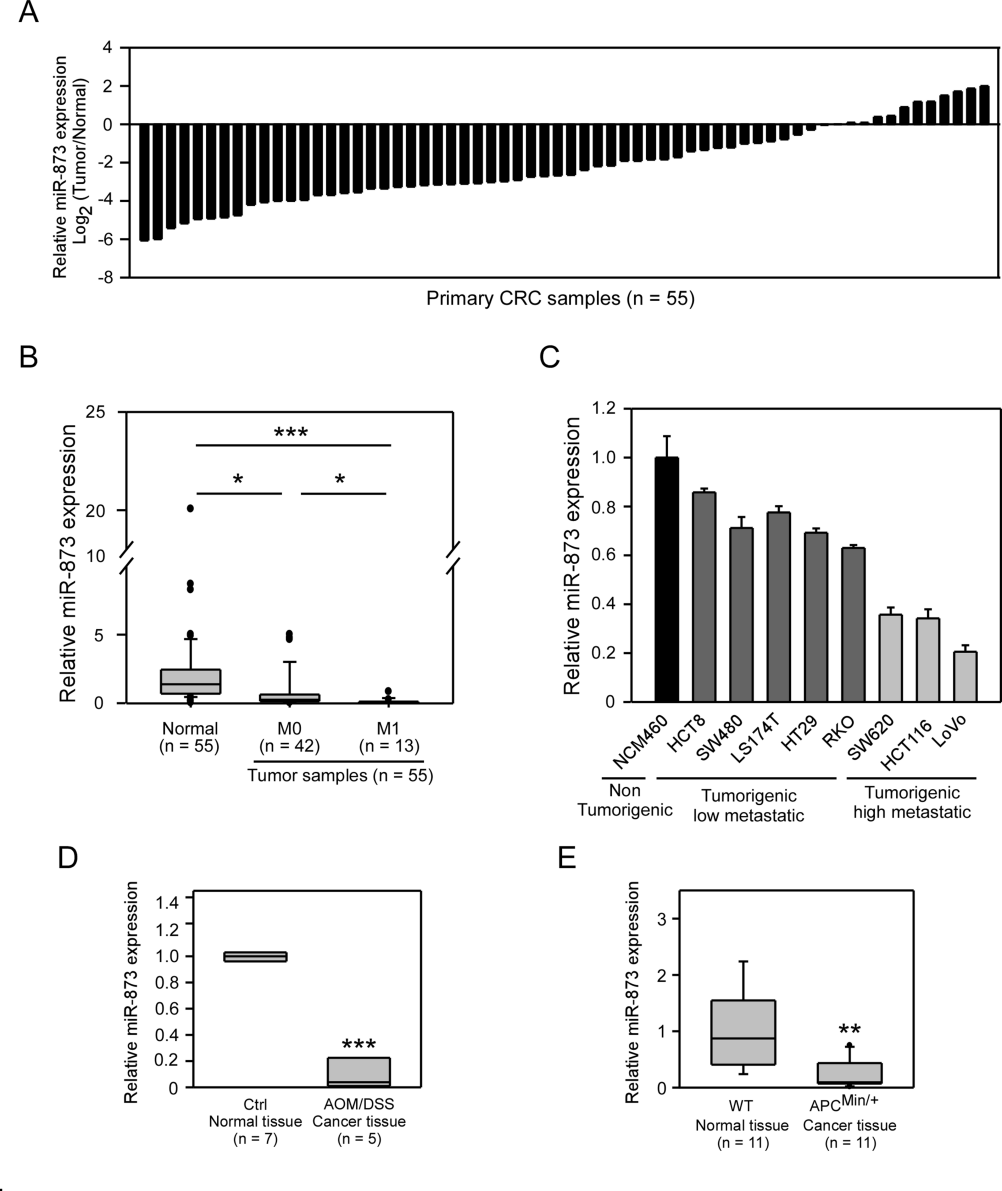
MiR-873 was downregulated in CRC clinical samples, mouse models and CRC cell lines. (**A**) qRT-PCR analysis of miR-873 levels in 55 paired CRC clinical specimens. (**B**) Corelation between miR-873 levels and the distant metastasis status of CRC samples. (**C**) qRT-PCR analysis of miR-873 levels in normal colon cell line and CRC cell lines with different metastatic potential. (**D**, **E**) qRT-PCR analysis of miR-873 expression in AOM/DSS mouse model (D) and *APC^Min+^* mouse model (E). Data (mean ± SEM) are representative of three technique replicates. ^*^P < 0.05; ^**^P < 0.01; ^***^P < 0.001.

To determine the clinical significance of miR-873 expression, we first detected the expression levels of miR-873 in paired human clinical samples of CRC. MiR-873 expression was downregulated in 43 of 55 (78.2%) CRC tumor specimens compared to their normal counterparts (Figure 1A). The miR-873 levels in CRC tumors (median = 0.60) were significantly lower (*P* < 0.001) than that in normal colon samples (median = 2.22). Moreover, in primary tumor samples of patients with liver metastases, miR-873 expression was even lower than those without liver metastases (Figure 1B). And, the relationships of miR-873 expression with clinicopathological factors of CRC was shown in Table 1. The decrease of miR-873 expression was found to be significantly related to distant metastasis. However, no significant correlations were found between miR-873 expression and other factors including age, gender, clinical stage and lymph node metastasis. Interestingly, miR-873 levels in CRC cell lines with high metastatic potential (SW620, HCT116 and LoVo) were significantly lower than those cell lines with low metastatic potential (HCT8, SW480, LS174T, HT29 and RKO) and normal colon epithelial cell line NCM460 (Figure 1C). The AOM/DSS mouse model is a colitis-associated CRC model and the *APC^Min+^* mouse model is a spontaneous CRC model. These two models can mimic most of the cases in human CRC progression [16–18]. We interrogated miR-873 expression in samples from these two kinds of mouse models. As shown in Figure 1D, miR-873 expression in tumor tissues from the AOM/DSS-administrated group was significantly lower than that in normal colon tissues from control group. Likewise, miR-873 expression was decreased in tumor tissues from *APC^Min+^* mice compared with normal colon tissues from wild type mice (Figure 1E). These data indicated that miR-873 may be a tumor suppressor and is negatively correlated with the metastatic potential of CRC.

**Table 1:** Relationships between miR-873 expression levels with clinicopathological factors in CRC

**Variables**	***N***	**miR-873**		***P***
		**Low**	**High**	
Age (years)				0.200
> 61	29	21	8	
≤ 61	26	24	2	
Gender				0.230
Male	35	27	8	
Female	20	18	2	
Clinical stage				0.190
I-II	16	12	4	
III-IV	39	33	6	
pN stage				0.057
N0	14	9	5	
N1-N2	41	36	5	
Distant metastasis				0.031^*^
No	42	33	9	
Yes	13	12	1	

^*^P < 0.05 by Student’s *t*-test

### Overexpression of miR-873 inhibits CRC cell proliferation, migration and invasion *in vitro*

**Figure 2:**
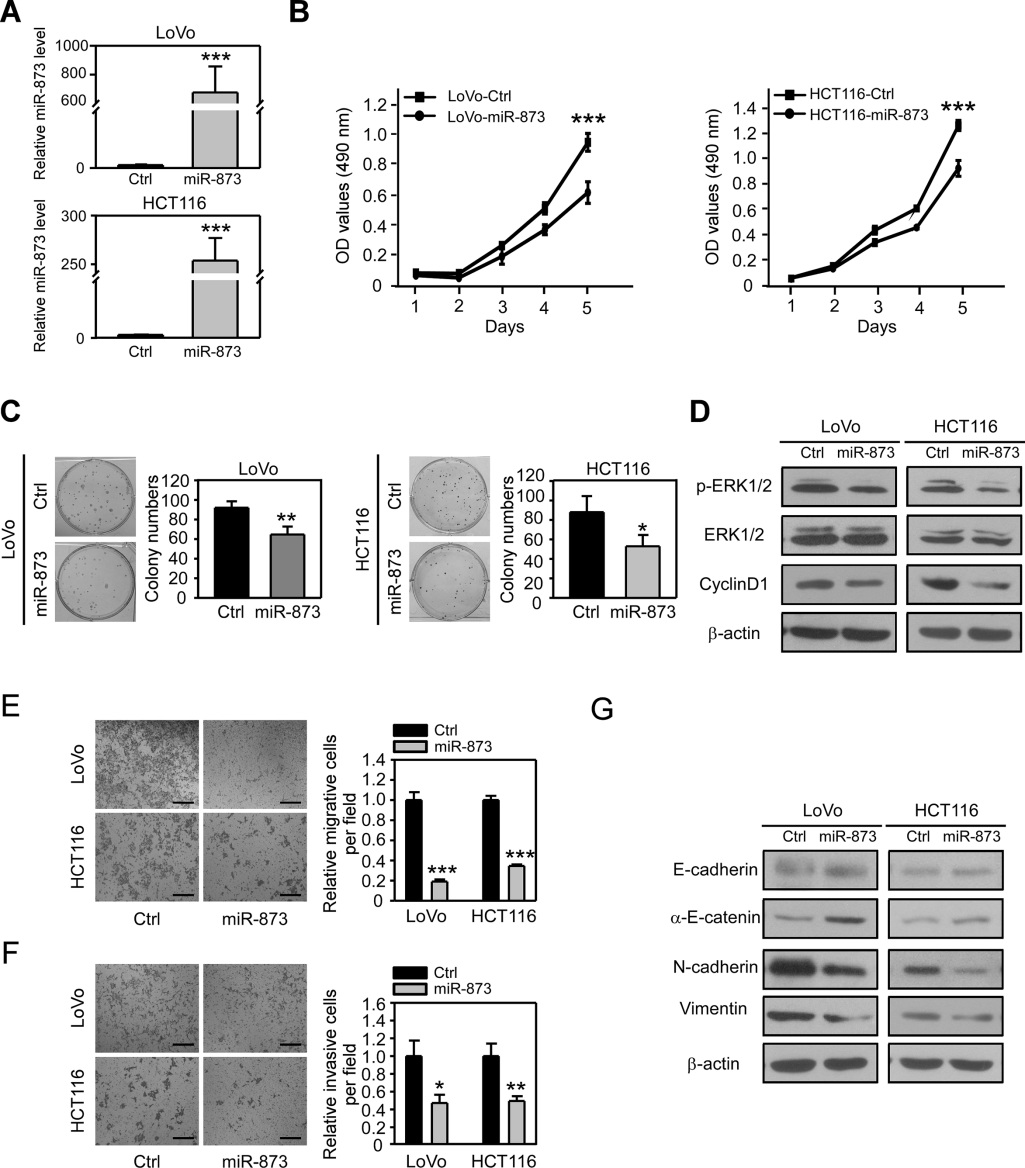
MiR-873 inhibits CRC cell proliferation, migration and invasion *in vitro*. (**A**) qRT-PCR analysis of miR-873 levels in LoVo and HCT116 stable infected cell lines. (**B**) MTT assay of LoVo-control and LoVo-miR-873 cells (left) and HCT116-control and HCT116-miR-873 cells (right). (**C**) Representative images and quantification of colonies formed by LoVo-control and LoVo-miR-873 cells (left) and HCT116-control and HCT116-miR-873 cells (right). (**D**) Western blotting analysis of p-ERK1/2, ERK1/2 and CyclinD1 in LoVo/HCT116-control and LoVo/HCT116-miR-873 cells. β-actin was used as a loading control. (**E**, **F**) Representative images and quantification of cell migration (E) and invasion (F) assays of LoVo/HCT116-control and LoVo/HCT116-miR-873 cells. (**G**) Western blotting analysis of EMT-related markers in LoVo/HCT116-control and LoVo/HCT116-miR-873 cells. β-actin was used as a loading control. Data (mean ± SEM) are representative of 3 technique replicates. Scale bars, 100 μm. ^*^P < 0.05; ^**^P < 0.01; ^***^P < 0.001.

To reveal the biological roles of miR-873 in CRC, we overexpressed pre-miR-873 in two CRC cell lines LoVo and HCT116 with high metastatic potential (Figure 2A). MTT assay was performed to examine the effect of miR-873 overexpression on CRC cell proliferation. As shown in Figure 2B, ectopic expression of miR-873 significantly inhibited proliferation of LoVo and HCT116 cells. Colony formation assays showed that miR-873 overexpressing cells formed less colonies than control cells (Figure 2C), which also indicated that miR-873 inhibits CRC cell proliferation *in vitro*. We further found that the phosphorylated level of ERK1/2 and the protein level of CyclinD1 were decreased upon overexpression of miR-873 in LoVo and HCT116 cells (Figure 2D). Therefore, we speculated that miR-873 may exert its tumor-suppressive role by affecting the ERK-CyclinD1 axis which is one of the most essential pathways that control cell proliferation [19]. Since miR-873 expression was shown to be negatively correlated with the metastatic ability in CRC clinical samples and cell lines, we further performed Transwell assays to examine whether miR-873 could inhibit migration and invasion of CRC cells. As a result, overexpression of miR-873 in LoVo and HCT116 cells dramatically suppressed their migration (Figure 2E) and invasion (Figure 2F). We further wanted to know how miR-873 elicits its metastasis-inhibitory function. EMT is a prominent process for tumor cells to dedifferentiate from an epithelial into a more mesenchymal phenotype, upon which they become more migratory and invasive and gain metastatic ability [20]. Therefore, we analyzed the expressions of epithelial markers E-cadherin and α-E-catenin as well as mesenchymal markers N-cadherin and Vimentin. Interestingly, ectopic expression of miR-873 made the expression of epithelial markers upregulated while expression of mesenchymal markers downregulated (Figure 2G). Altogether, these results indicated that miR-873 could inhibit CRC cell proliferation, migration and invasion *in vitro* significantly.

### Inhibition of miR-873 promotes CRC cell proliferation, migration and invasion *in vitro*

**Figure 3:**
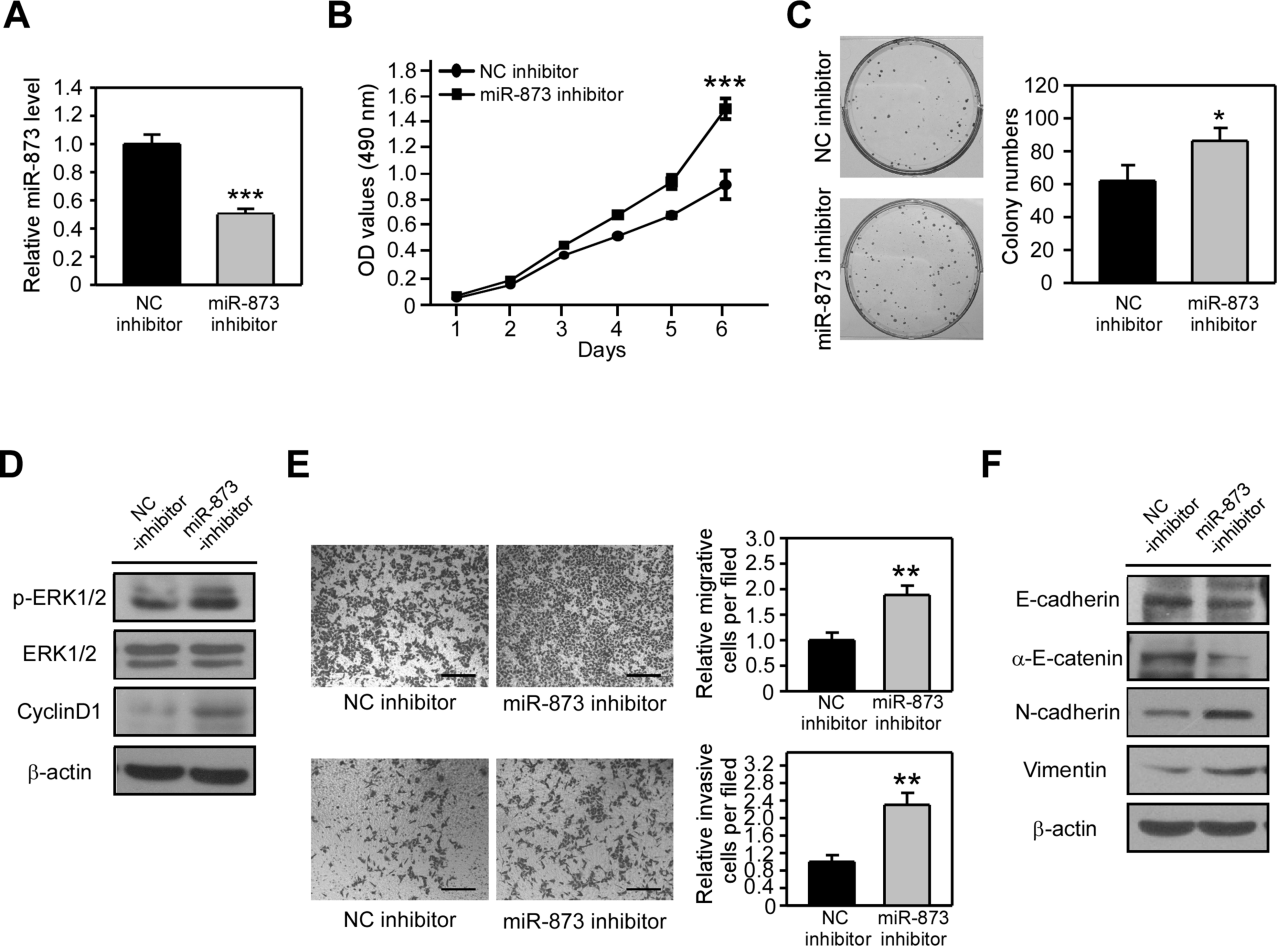
MiR-873 inhibition promotes CRC cell proliferation, migration and invasion *in vitro*. (**A**) qRT-PCR analysis of miR-873 levels in HCT8 cells transfected with negative control inhibtor (HCT8-NC inhibitor) and miR-873 inbihitor (HCT8-miR-873 inhibitor). (**B**) MTT assay of HCT8-NC inhibitor and HCT8-miR-873 inhibitor cells. (**C**) Representative images and quantification of colonies formed by HCT8-NC inhibitor and HCT8-miR-873 inhibitor cells. (**D**) Western blotting analysis of p-ERK1/2, ERK1/2 and CyclinD1 in HCT8-NC inhibitor and HCT8-miR-873 inhibitor cells. (**E**) Representative images and quantification of cell migration and invasion assays of HCT8-NC inhibitor and HCT8-miR-873 inhibitor cells. (**F**) Western blotting analysis of EMT-related markers in HCT8-NC inhibitor and HCT8-miR-873 inhibitor cells. Data (mean ± SEM) are representative of 3 technique replicates. Scale bars, 100 μm. ^*^P < 0.05; ^**^P < 0.01; ^***^P < 0.001.

Because miR-873 overexpression suppressed CRC cell proliferation, migration and invasion, we wondered if inhibition of miR-873 can augment CRC cell proliferation and motility. We applied a miR-873 inhibitor to block endogenous miR-873 expression in HCT8 cells (Figure 3A) whose metastatic ability is relative lower and its miR-873 level is the highest among all the CRC cell lines we used (Figure 1C). When miR-873 level was decreased, cell proliferation rate was significantly increased as detected by MTT assay (Figure 3B) and colony formation assay (Figure 3C). Moreover, the phosphorylated level of ERK1/2 and the protein level of CyclinD1 were increased after miR-873 inhibition (Figure 3D). We subsequently explored whether miR-873 knockdown could increase the proliferation, migration and invasion of HCT8 cells. As shown in Figure 3E, cell migration and invasion were dramatically strengthened upon miR-873 inhibition. Moreover, protein levels of E-cadherin and α-E-catenin were downregulated and the levels of N-cadherin and Vimentin were upregulated in HCT8 cell transfected with miR-873 inhibitor (Figure 3F). Collectively, our loss-of-function data indicated that inhibition of miR-873 result in the promotion of CRC cell proliferation, migration and invasion.

### Overexpressing miR-873 suppresses CRC cell growth and liver metastasis *in vivo*

**Figure 4:**
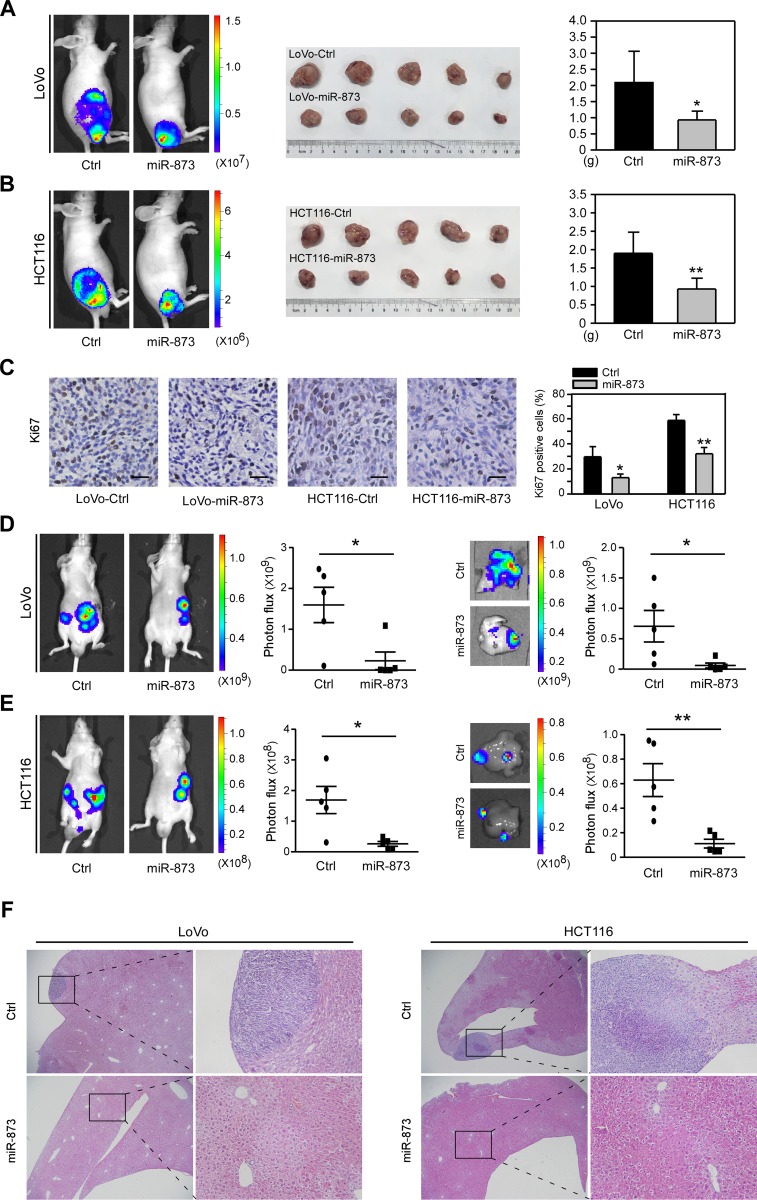
MiR-873 suppresses CRC cell growth and liver metastasis *in vivo*. (**A**) Images of bioluminescence of mice that was subcutaneously injected with LoVo-control and LoVo-miR-873 cells (left). Image (middle) and weight quantification (right) of subcutaneous tumors formed by LoVo-control and LoVo-miR-873 cells. (**B**) Images of bioluminescence of mice that was subcutaneously injected with HCT116-control and HCT116-miR-873 cells (left). Image (middle) and weight quantification (right) of subcutaneous tumors formed by HCT116-control and HCT116-miR-873 cells. (**C**) Representative images of IHC staining and quantification of Ki67-positve cells rate in tumors formed by LoVo/HCT116-control and LoVo/HCT116-miR-873 cells. (**D**) Images and quantification of bioluminescence imaging of mice (left) and livers (right) intrasplenically injected with LoVo-control and LoVo-miR-873 cells. (**E**) Images and quantification of bioluminescence imaging of mice (left) and livers (right) intrasplenically injected with HCT116-control and HCT116-miR-873 cells. Scale bars, 100 μm. (**F**) H&E of livers intrasplenically injected with LoVo-control and LoVo-miR-873 cells or HCT116-control and HCT116-miR-873 cells. Original magnification, ×40 (left of each cell), ×200 (close-up, right of each cell) ^*^P < 0.05; ^**^P < 0.01.

The above results revealed that miR-873 overexpression could inhibit cell proliferation, migration and invasion *in vitro*, which prompted us to test whether it can affect these traits *in vivo*.We first infected LoVo and HCT116 cells with a lentivirus that could stably overexpress a *Luciferase* gene, followed by infecting these two Luciferase-labeled cells with lentiviruses encoding the vector or pre-miR-873. Then, stable infected LoVo and HCT116 cells were subcutaneously injected into nude mice and bioluminescence imaging was performed after 4 weeks. As shown in Figure 4A, LoVo cells with miR-873 overexpression formed smaller tumors compared with control cells. We then isolated the xenograft tumors and found the weight of LoVo-miR-873 tumors was significantly decreased compared with LoVo-Control tumors (Figure 4A). Similarly, we observed ectopic expression of miR-873 in HCT16 cells also dramatically suppresses tumor growth *in vivo* (Figure 4B). And then, the expression of proliferation marker Ki67 in the isolated tumors was further detected. The proportion of Ki67-positive cells in tumors formed by miR-873 overexpressing cells were much lower than that in tumors formed by control cells (Figure 4C). Liver is the most vital target organ for metastatic CRC and liver metastasis is the direct cause of CRC death [21]. Thus, we further assessed the metastatic ability of miR-873-overexpressing cells by injecting them into nude mice intrasplenically to construct an experimentally metastatic model. Bioluminescence imaging results showed that LoVo (Figure 4D) and HCT116 (Figure 4E) cells with miR-873 overexpression formed less hepatic metastatic nodules which were validated by H&E staining of liver slices (Figure 4F). In summary, these above results indicated that miR-873 could inhibit CRC cell growth and metastasis *in vivo*.

### ELK1 and STRN4 are direct targets of miR-873

**Figure 5:**
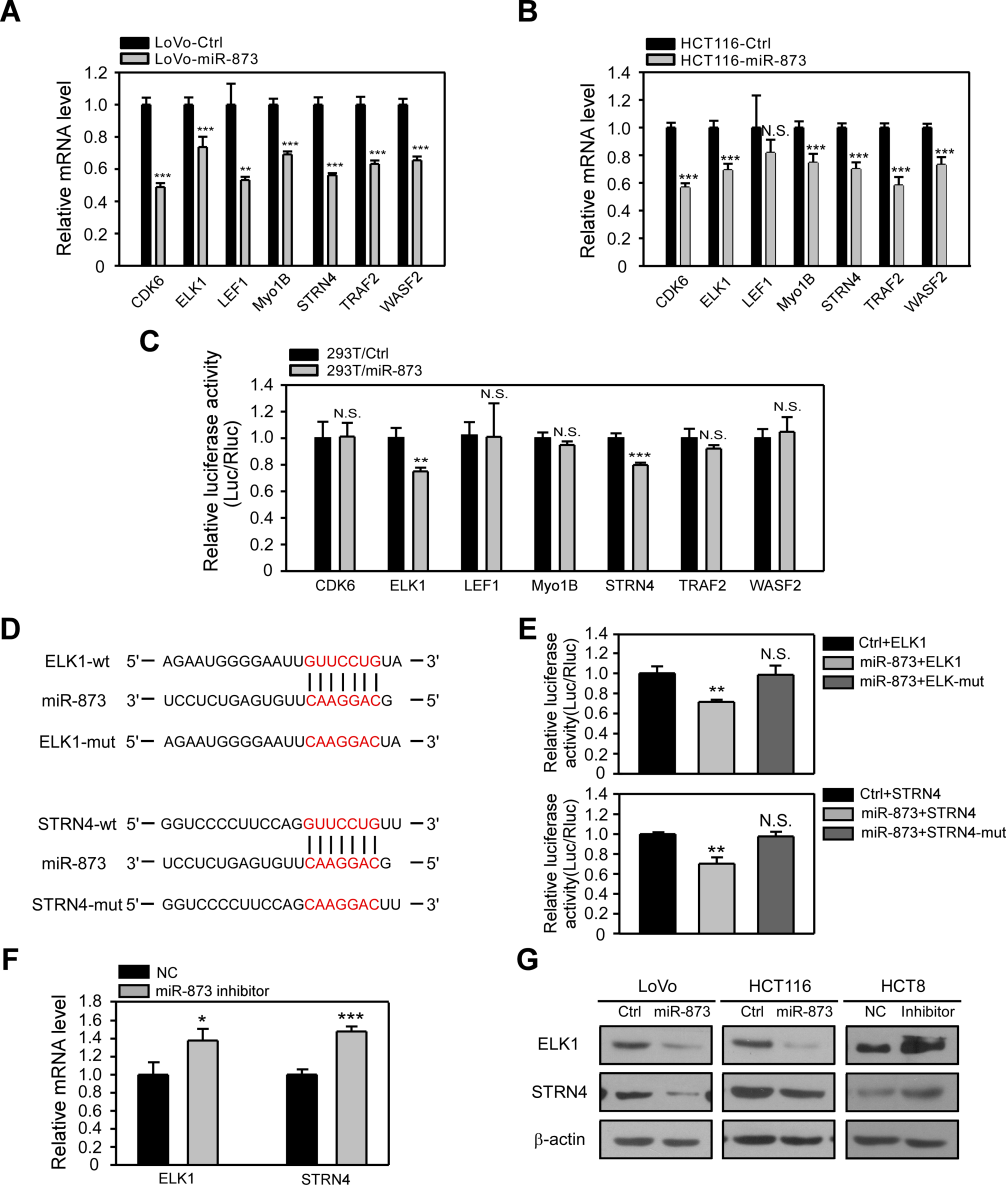
ELK1 and STRN4 are direct targets of miR-873. (**A**, **B**) qRT-PCR analysis of mRNA levels of candidate genes in LoVo-control and LoVo-miR-873 cells (A) and HCT116-control and HCT116-miR-873 cells (B). (**C**) Dual Luciferase reporter assays of 3ʹUTRs of candidate genes upon transfection of miR-873 in 293T cells. (**D**) Schematic of mutations generetated in miR-873 binding regions in 3ʹUTRs of ELK1 and STRN4. (**E**) Dual Luciferase reporter assays of wild-type or mutant 3ʹUTRs of candidate genes upon transfection of miR-873 in 293T cells. (**F**) qRT-PCR analysis of ELK1 and STRN4 mRNA levels in HCT8-NC inhibitor and HCT8-miR-873 inhibitor cells. (**G**) Western blotting analysis of ELK1 and STRN4 protein levels in LoVo/HCT116-control, LoVo/HCT116-miR-873 cells, HCT8-NC inhibitor and HCT8-miR-873 inhibitor cells. ^*^P < 0.05; ^**^P < 0.01; ^***^P < 0.001; N.S. no significance.

MiRNAs exert their biological roles by targeting the 3ʹUTR of mRNAs, resulting in mRNA degradation and/or translational inhibition. On the basis of above results which implied miR-873 may serve as a tumor-suppressive miRNA, we further aimed to identify its targets. Therefore, we applied the most common *in silico* prediction algorithm TargetScan to mine the candidate targets of miR-873. And we selected seven oncogenes that have been proved to affect cell proliferation or/and mobility. The mRNA levels of CDK6, ELK1, MyoB1, STRN4, TRAF2 and WASF2 were revealed to be significantly decreased after ectopic expression of miR-873 in both LoVo and HCT116 cells (Figure 5A and 5B). Moreover, we performed Dual Luciferase Reporter Assay to verify whether there were direct interactions between miR-873 and these genes. MiR-873 and the 3ʹUTR elements of these genes were co-transfected into 293T cells. As showed in Figure 5C, overexpressing miR-873 led to the decrease of Luciferase activity of the 3ʹUTRs of ELK1 and STRN4 rather than 3ʹUTRs of CDK6, MyoB1, TRAF2 and WASF2. However, suppression of Luciferase activity was abolished when the putative binding sequences of miR-873 were mutated (Figure 5D and 5E). The mRNA levels of ELK1 and STRN4 in HCT8 cells transfected with miR-873 inhibitor was further detected. Upon miR-873 inhibition, ELK1 and STRN4 showed higher mRNA levels (Figure 5F). Consistent with the qRT-PCR results, the protein levels of ELK1 and STRN4 were decreased in LoVo and HCT116 cells overexpressing miR-873 while their expression was increased in HCT8 cells transfected with the miR-873 inhibitor (Figure 5G). These data demonstrated that miR-873 could block ELK1 and STRN4 expression in CRC cells by binding to their 3ʹUTRs.

### Restoration of ELK1 and STRN4 can partially rescue phenotypes restrained by miR-873

**Figure 6:**
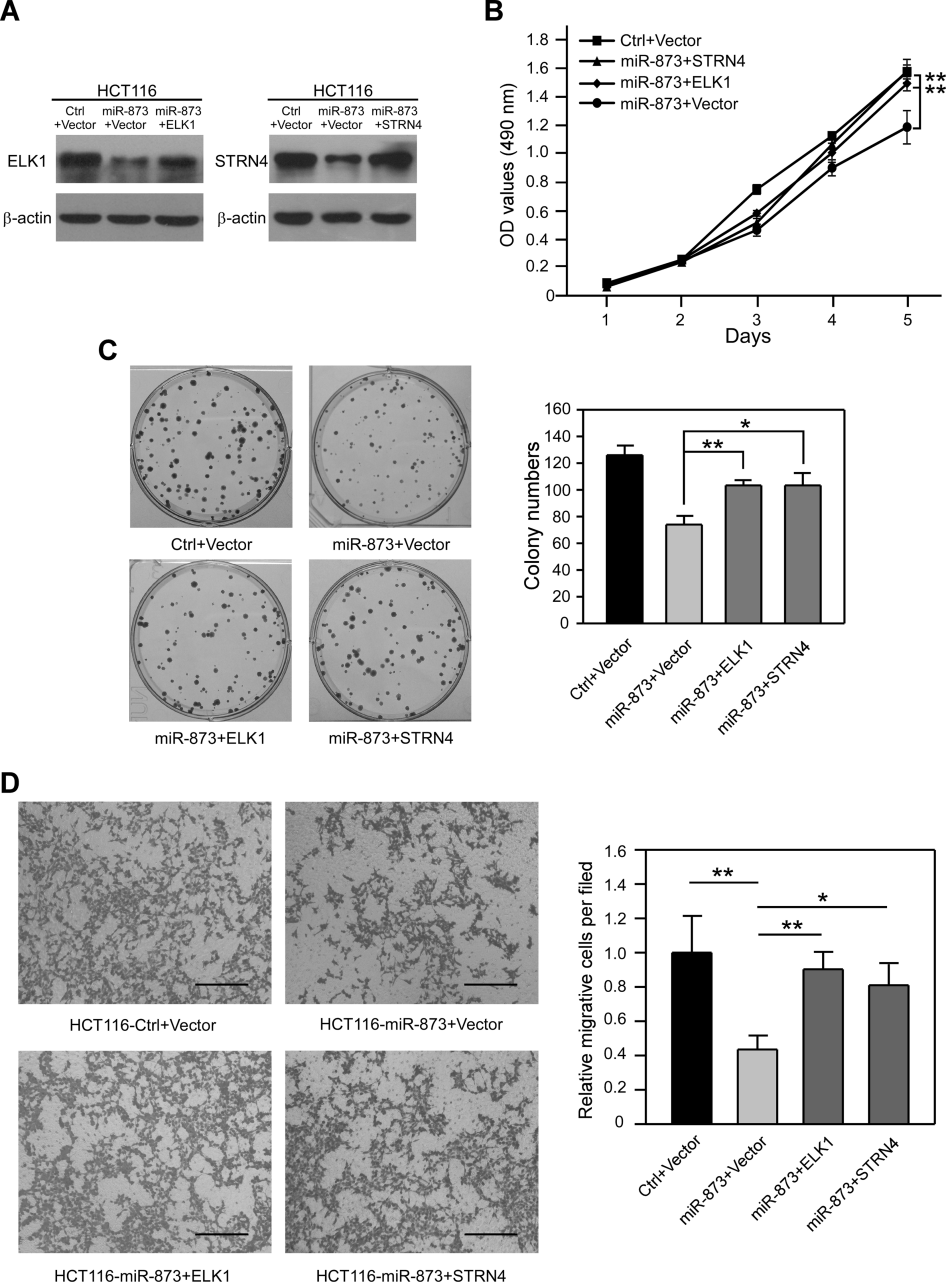
Re-activation of ELK1 and STRN4 can partially rescue phenotypes restrained by miR-873. (**A**) Western blotting analysis of ELK1 and STRN4 protein levels in HCT116-control and HCT116-miR-873 cells re-expressing ELK1 or STRN4. (**B**) MTT assays of HCT116-control and HCT116-miR-873 cells re-expressing ELK1 or STRN4. (**C**) Representative images and quantification of colonies formed by HCT116-control and HCT116-miR-873 cells re-expressing ELK1 or STRN4. (**D**) Representative images and quantification of cell migration and invasion assays of HCT116-control and HCT116-miR-873 cells re-expressing ELK1 or STRN4. Data (mean ± SEM) are representative of 3 technique replicates. Scale bars, 100 μm. ^*^P < 0.05; ^**^P < 0.01.

To further estimate whether ELK1 and STRN4 are vital downstream targets of miR-873, we analyzed the proliferation and migration of CRC cells through restoration of ELK1 and STRN4 expression. To this end, miR-873-overexpressing HCT116 cells were transfected with plasmids encoding ELK1 and STRN4 which lack 3ʹUTR elements, and their protein levels were recovered (Figure 6A). After restoring ELK1 and STRN4, MTT and colony formation assays showed that the inhibitory effects of miR-873 on HCT116 cell proliferation were obviously alleviated (Figure 6B and 6C). Moreover, re-activation of ELK1 and STRN4 in HCT116 cells partially reversed the suppression of migratory ability due to miR-873 overexpression (Figure 6D). Taken together, we indicated that miR-873 decreases the proliferation and motility of CRC cells at least partially by targeting ELK1 and STRN4.

## DISCUSSION

MiRNAs are a large class of non-coding RNAs which have been well proved to function as modulators in tumorigenesis and cancer progression [22, 23]. As a very common malignancy, CRC was reported to be widely regulated by miRNAs. MiR-1269a, a miRNA activated by TGF-β, promotes TGF-β signaling via targeting Smad7 and HOXD10, thereby forming a positive feedback loop and increases invasion and metastasis of CRC cells [24]. Recently, we revealed that miR-543 acts as a onco-suppressor miRNA by restraining the RAS-MEK-ERK-CyclinD1 axis for growth inhibition and targeting HMGA2 and MTA1 for mobility suppression [9]. To date, however, roles of miR-873 in CRC are yet unknown. In the present study, we found that miR-873 level was significantly decreased in CRC patient specimens, especially in samples with metastatic potential. Notably, there was a negative correlation between miR-873 expression and the metastatic ability of CRC cell lines. Gain-of-function experiments revealed that proliferation, migration and invasion of CRC cells were suppressed after ectopic expression of miR-873 *in vitro* and *in vivo*. In contrast, miR-873 depletion dramatically promoted CRC cell growth and motility *in vitro*. Furthermore, miR-873 overexpression decreased the phosphorylation of ERK1/2 and the level of CyclinD1, which may elicit the proliferation blockage. The EMT process, a prominent driver for tumor metastasis, was also retardant upon overexpression of miR-873. To find the direct targets of miR-873, we combined an *in silico* prediction tool with functional validation experiments. Ultimately, we identified ELK1 and STRN4 as downstream target genes for miR-873 to exert its tumor-suppressive roles.

MiR-873 has been demonstrated to play diverse roles in various types of cancers. It is first identified to suppress breast cancer cell proliferation and enhance tamoxifen resistance by targeting CDK3 [13]. In glioma cells, miR-873 overexpression results in the decrease of Bcl-2, followed by suppressing cell proliferation, motility and promoting apoptosis in cells which are cisplatin resistant [25]. Similarly, miR-873 acts as an apoptosis sensitizer of ovarian cells by targeting ABCB1 [12]. Futhermore, IGF2BP1 has been proved to be a target of miR-873 in glioblastoma cells and their growth and metastasis are inhibited by miR-873 [11]. However, miR-873 has been reported as an oncogene in lung adenocarcinoma by inhibiting its downstream target SRCIN1 [14]. The multiple functions of miR-873 in different cancer types may depend on the specific cellular contexts. Previous study indicated the hypermethylation of miR-873 promoter is a common event in CRC cell lines. Consistently, our qRT-PCR results revealed miR-873 was downregulated in CRC patient samples. It is worth noting that in addition to hypermethylation, other regulatory mechanisms involved in the dysregulation of miR-873 in CRC need further investigation.

ELK1 is a crucial transcription factor that mediates the MEK-ERK signaling transduction. It functions as an activator for early oncogene expression like c-Fos when tumor cells sense various extracellular signals and promotes cell proliferation [26, 27]. Moreover, ELK1 plays a key role in eliciting EMT in non-small cell lung cancer (NSCLC) and gastric cancer cells [28, 29]. STRN4 is a member of the striatin family which forms a large complex with proteins of the germinal center kinase family [30]. It is highly expressed in a variety of cancer cells including HCT116 and knockdown of STRN4 dramatically decreases proliferation and migration of HCT116 cells [31]. Our data indicated that miR-873 directly binds and inhibits ELK1 and STRN4, thereby suppressing growth and metastasis of CRC cells. Given the multi-targeting trait of miRNA, there may be other downstream targets that mediate the tumor-suppressive role of miR-873.

We used samples from CRC patients and two CRC mouse models to test the diagnostic and prognostic values of miR-873. Although our results showed that low miR-873 level may be related to CRC progression, further investigations that employ a much larger-scale approach are required to determine whether it could serve as a reliable biomarker for clinical detection. Collectively, our study highlights the novel roles of miR-873 in alleviating malignant proliferation and metastasis of CRC cells.

## MATERIALS AND METHODS

### Clinical specimens

55 paired CRC samples and their normal counterparts were acquired from the Department of Surgical Oncology, First Affiliated Hospital of Xiamen University (Xiamen, China). Tissues were immediately put into Liquid Nitrogen after surgery and stored at −80°C until RNA isolation. Experiments were performed in according to the guidelines by the Ethics and Scientific Committees of Xiamen University with consent of the patients.

### Cell culture

CRC cell lines SW620 and HCT116 were kindly provided by Dr. Han You (School of Life Sciences, Xiamen University). HCT8, SW480, LS174T, HT29 and RKO were obtained from the Institute of Biochemistry and Cell Biology, Chinese Academy of Sciences, Shanghai. 293T cells were obtained from ATCC. HCT116 cells were maintained in McCoy’s 5A supplemented with 10% fetal bovine serum (FBS). 293T, SW620, LS174t and HT29 cells were cultured in DMEM supplemented with 10% FBS. HCT8, SW480, RKO and LoVo cells were maintained in RPMI-1640 media supplemented with 10% FBS.

### Animal studies

*APC^Min+^* mice were kindly provided by Dr. Jiahuai Han (School of Life Sciences, Xiamen University). The colitis-associated CRC mouse model was constructed as previously described [9]. For *in vivo* cell growth assays, 5 × 10^6^ cells were subcutaneously injected into the lower back region of 6–8 weeks-old male nude mice for 28 days (*n* = 5 per group). For experimental liver metastasis model, 5 × 10^5^ cells were intrasplenically injected into the 6–8 weeks-old male nude mice for 28 days (*n* = 5 per group). Bioluminescence imaging was performed in the live animal Lumina II system (Xenogen IVIS system).

### Plasmid construction, cell transfection and infection

Plasmid construction was done as previously described [32]. The Has-pre-miR-873 flank region was inserted into pCDH-CMV-EF1α-puro vector and 3ʹUTR regions containing the predicted miR-873 binding sites were amplified and inserted into the pMIR-REPORT Vector. Primers used for plasmid construction were listed in Supplementary Table 1. For generation of stable cell lines, a lentivirus-based packaging system (pMDL, REV and VSVG) was used [33, 34]. MiR-873 inhibitor and the negative control inhibitor were designed and synthesized by Genepharma (Shanghai, China). For cell transfection, Lipofectamine 2000 (Invitrogen) was used according to the manufacturer’s instruction. *In vitro* experiments were performed and total RNA and protein were isolated 48 hours after transfection.

### Cell proliferation assays

For MTT assay, 2 × 10^3^ cells were seeded in 96-well plates. MTT was added to the medium and the absorbance at 490 nm was tested every day until the 5^th^ day. For colony formation assay, 300 cells were seeded in 6-well plates. After two weeks, cells were fixed, stained and photographed. Experiments were performed in triple.

### Cell migration and invasion assays

Transwell plates (Corning) containing 8 μm-pore size membranes were used for migration (without matrigel) or invasion (with matrigel) assays. For migration assays, 5 × 10^4^ LoVo, HCT116 cells or HCT8 cells were seeded in the top chamber. For invasion assays, 1 × 10^5^ LoVo, HCT116 cells or HCT8 cells were seeded in the top chamber. After 48 hours, cells on the lower flat were fixed, stained and photographed. Experiments were performed at least for three times.

### Dual Luciferase reporter assays

MiR-873-overexpressing plasmid or vector and 3ʹUTR-Luciferase plasmids were co-transfected into 293T cells seeded in a 24-well plate. Cell were lysed 48 hours after transfection and activities of Luciferase and Rellina were measured by the Dual-Glo Luciferase Assay System (Promega). Experiments were performed for three times.

### Quantitative real-time PCR (qRT-PCR)

Cell or tissues were lysed by the Trizol reagent (Roche) and total RNA was isolated. TaqMan MicroRNA Reverse Transcription Kit (ABI) was used for miRNA reverse transcription. ReverTra Ace qPCR RT Kit (Toyobo) was applied for mRNA reverse transcription. TransStart Top Green qPCR SuperMix (TransGen Biotech) was used for quantification of miRNA/mRNA level. GAPDH (for mRNA) and U6 (for miRNA) were used respectively as positive control and the 2^-ΔΔCt^ algorithm was applied for relative quantification of mRNA and miRNA levels. Primers used for qRT-PCR were listed in Supplementary Table 2.

### Western blotting

Western blotting was performed as previously described [32]. Immunoblot was carried using the following primary antibodies: ELK1 and STRN4 (Abcam); Vimentin (R&D); CyclinD1 (Santa Cruz); E-Cadherin and N-Cadherin (BD); α-E-catenin, ERK1/2 and p-ERK (Cell Signaling); β-actin (Thermo Fisher).

## SUPPLEMENTARY MATERIALS FIGURES AND TABLES



## Abbreviations

3ʹUTR: 3ʹ-untranslated-region
AOM/DSS: azoxymethane/dextran sodium sulfate
CDK6: cyclin dependent kinase 6
ELK1: ETS transcription factor
MyoB1: myosin IB
STRN4: striatin 4
TRAF2: TNF receptor associated factor 2
WASF2: WAS protein family member 2
NSCLC: non-small cell lung cancer
MTT: 3-(4,5-dimethyl-2-thiazolyl)-2,5-di-phenyl-2-H-tetrazolium bromide
FBS: fetal bovine serum
