# Supplementary material for: MicroRNA-873 inhibits colorectal cancer metastasis by targeting ELK1 and STRN4
Source: Oncotarget. 2018 Jan 2;10(41):4192–204. doi: 10.18632/oncotarget.24115 (PMC6609243; doi:10.18632/oncotarget.24115)
Supplement: Supplementary file 1 [file oncotarget-10-4192-s001.pdf]

MicroRNA-873 inhibits colorectal cancer metastasis by targeting ELK1 and STRN4

SUPPLEMENTARY MATERIALS

Supplementary Table 1: Primer sequences for plasmid construction

pre-miR-873	Fw	AGTGTCTGGGATGCCCACAA
	Rv	TGAGTGGACAAGTTAACCCTTGC
CDK6 3’UTR	Fw	ATACTCATCTAGGCTGTGTGAACC
	Rv	GTGTGGCCTGTAGATGGAGAGA
ELK1 3’UTR	Fw	ATGTCCCTGGAGTTGGGAGG
	Rv	CACAGCTTTCCACGTCTCTGAC
LEF1 3’UTR	Fw	CCTTGTAAGGTGTTCAGTAGAGCT
	Rv	CACTCCCATCTGAATCCTGGC
MyO1B 3’UTR	Fw	GGTGATTGGTACAGGGTGCC
	Rv	CTGAGAGATGATACATTGACACACAAC
STRN4 3’UTR	Fw	AGGGATTGCCCAACACTCCT
	Rv	TTTCACATACAAGATGGGGACAGA
WASF2 3’UTR	Fw	GTGTTCAGGATGCACCACCA
	Rv	AGGCTGGTCCCAAGGCTATG
ELK1 CDS	Fw	ATGGACCCATCTGTGACGC
	Rv	TCATGGCTTCTGGGGCC
STRN4 CDS	Fw	ATGATGGAGGAGCGAGCG
	Rv	TCATACGAAGACCTTGGCCA
ELK1 3’UTR mut	Fw	ATTCAAGGACTAAATATATATTTTTCAAAGTGATGCTGGAG
	Rv	CTTTAGTCCTTGAATTCCCCATTCTCTGGGACTC
STRN4 3’UTR mut	Fw	CAGCAAGGACATTATAAGCCCCAACCCCTCTG
	Rv	TATAATGTCCTTGCTGGAAGGGGACCGAGGG

Supplementary Table 2: Primer sequences for qRT-PCR analysis

miR-873 RT		TCGTATCCAGTGCGTGTCGTGGAGTCGGCAATTGCACTGGATACGACT AGGAGA
U6 RT		CGCTTCACGAATTTGCGTGTCAT
miR-873 qPCR	Fw	GGGGGCAGGAACTTGTGAG
	Rv	TGCGTGTCGTGGAGTC
U6 qPCR	Fw	GCTTCGGCAGCACATATACTAAAAT
	Rv	CGCTTCACGAATTTGCGTGTCAT
CDK6	Fw	TGCACAGTGTCACGAACAGACA
	Rv	ACCTCGGAGAAGCTGAAACATCA
ELK1	Fw	CTTCTGGAGCACCCTGAGTCC
	Rv	GAGAGCATGGATGGAGTGACC
LEF1	Fw	TGTTTATCCCATCACGGGTGG
	Rv	CATGGAAGTGTCGCCTGACAG
Myo1B	Fw	AGGTCTGGTGTGGAGGTCCTAT
	Rv	GCGTTGCTTCCTCAGGTCTTC
STRN4	Fw	GGGAGTCGCTGCTGGTGAAA
	Rv	ATCGTCCTCGTCGCTGTCTTC
WASF2	Fw	CAAGACACGTAAGGAAGAGTGGG
	Rv	CACTGGGTAACTGAATTCTGCTGG
GAPDH	Fw	GGTCTCCTCTGACTTCAACA
	Rv	GTGAGGGTCTCTCTCTTCCT
